# Visuospatial asymmetries do not modulate the cheerleader effect

**DOI:** 10.1038/s41598-018-20784-5

**Published:** 2018-02-07

**Authors:** Daniel J. Carragher, Blake J. Lawrence, Nicole A. Thomas, Michael E. R. Nicholls

**Affiliations:** 1 College of Education, Psychology, and Social Work, Flinders University, Adelaide, Australia; 2 Ear Science Institute Australia, Subiaco, Australia; 3 Ear Sciences Centre, School of Surgery, The University of Western Australia, Perth, Australia; 4 Present Address: College of Healthcare Sciences, James Cook University, Cairns, Australia

## Abstract

The cheerleader effect occurs when the same individual appears to be more attractive when seen in a group, compared to alone. As observers over-attend to visual information presented in the left visual field, we investigated whether the spatial arrangement of the faces in a group would influence the magnitude of the cheerleader effect. In Experiment 1, *target faces* were presented twice in the centre of the display: once alone, and once in a group. Group images featured two *distractor faces*, which were presented in either the left or the right visual field, or on either side of the target. The location of the *distractor faces* did not modulate the size of the cheerleader effect, which was observed in each group configuration. In Experiment 2, we manipulated the location of the *target faces*, which were presented at the far left, far right, or centre of the group. Faces were again significantly more attractive in each group configuration, and the spatial location of the *target face* did not influence the size of the cheerleader effect. Together, our results show that the cheerleader effect is a robust phenomenon, which is not influenced by the spatial arrangement of the faces in the group.

## Introduction

Attractiveness is an important social cue that is rapidly evaluated from the face during first impressions^1,2^. Attractive individuals are attributed many positive stereotypes^3^, including competence^4^ and intelligence^5^. Furthermore, attractive individuals receive more lenient criminal sentences^6^, and an increased vote share in elections^7^, when compared to unattractive individuals. Facial attractiveness is signalled by the characteristics of the face being examined, including averageness, symmetry, and a sexually dimorphic appearance^8–10^. Because attractiveness is related to physical cues in the face, the majority of research has presented facial stimuli in isolation (i.e., a single face is presented at a time)^11^. Yet, we often meet strangers for the first time in social settings (e.g., in a boardroom or a bar). Recent findings have suggested that the perceived attractiveness of a face is influenced by social context^12–15^.

Previous research has shown that the presence of other faces in a group influences the attractiveness evaluations made for individual faces^13^. For example, the attractiveness of an individual is raised in the presence of an attractive group, but lowered in an unattractive group^15^. Furthermore, an unattractive, but task irrelevant, face can strongly influence the rate of preference choices made between two attractive faces^12^. Most curiously, Walker and Vul found that the same face is perceived to be more attractive when it is seen in a group, compared to when seen alone; a phenomenon described as ‘the cheerleader effect’^13,16^. The cheerleader effect occurs for both male and female faces, shown in groups of same- or mixed-gender faces. Furthermore, the cheerleader effect occurs for groups of various sizes, from 4–16 group members^13^. The cheerleader effect strongly suggests that it is not only the attractiveness of the individual face that is evaluated, but that the surrounding faces are also encoded by the observer, which interfere with attractiveness evaluations^11^. Together, these findings show that attractiveness judgments change when an individual appears in a group, and that the social perception of an individual within a group is a unique process, whereby irrelevant faces influence our judgments of specific individuals^11^.

When meeting a group for the first time, each group member is evaluated^11^. However, if each group member was evaluated individually, group perception would be both time consuming and cognitively demanding to perform. Rather, through the process of ensemble coding, the visual system rapidly summarises the group display, which allows observers to identify the mean characteristics of the group^17^. Through ensemble coding, observers are able to accurately report the average size of a group of circles^18^, or the average emotion displayed by a group of faces^19,20^. Although observers can accurately recall the average size of a group of circles^18^, when asked to recall the size of an individual circle from the group, observers recall the circle as being similar in size to the group average^21^. For example, a small individual circle presented among a group of large circles is recalled as being larger than it truly was. Brady and Alvarez^21^ suggest that ensemble coding occurs hierarchically, such that the average characteristics of the group influence the recall of individual items from the same group.

Walker and Vul^13^ proposed that the cheerleader effect occurs due to the hierarchical nature of ensemble coding. Initially, faces presented in a group image are automatically summarised into an ensemble average, through ensemble coding^22^. The ensemble average has the average characteristics of the faces in the group^20^, including the face being evaluated, and the irrelevant faces. Crucially, averageness is a trait that is perceived to be highly attractive in faces^8,23–25^. Average faces that are created by digitally averaging many faces together are perceived to be more attractive than the individual faces included in the averaging process^23,26^. Walker and Vul^13^ suggest that the ensemble average for a group of faces is also perceived to be highly attractive, because it has the average facial characteristics of the individual faces in the group. Walker and Vul suggest that the hierarchical structure of ensemble coding gives rise to the cheerleader effect, because an observer will recall the attractiveness of an individual face from the group as being similar to that of the ensemble average. Because the ensemble average is perceived to be highly attractive, observers will systematically recall any individual face seen in the group as being more attractive than when previously seen alone^13^.

The cheerleader effect demonstrates that irrelevant faces in the group (i.e., those not being evaluated), influence the perceived attractiveness of an individual. When considering how a group of individuals is commonly seen, it is clear that most groups are arranged horizontally so that the group members are standing side by side. The spatial arrangement of the faces within the group may modulate the strength of the cheerleader effect, because most people over-attend to visuospatial information that is presented within the left visual field (LVF); a phenomenon known as pseudoneglect^27,28^. This LVF bias likely arises because the right hemisphere, which processes the visual information in the LVF, is dominant for visuospatial processing^29^. Pseudoneglect is demonstrated in line bisection tasks, whereby observers erroneously mark the centre of a horizontal line to the left of the true centre^28,30^. Interestingly, pseudoneglect also influences representational memory^31^, whereby observers show greater accuracy when recalling landmarks that are seen in the LVF compared to the right visual field (RVF)^32^. Similarly, observers are more accurate in recalling changes in complex visual patterns when they occur in the LVF as opposed to the RVF^33^. The cheerleader effect might be modulated by the spatial arrangement of the faces in the group, because the attention of the observer is not equally distributed across the visual field, and consequently, the individual faces in the group.

Attentional asymmetries have also been shown to influence the processing of human faces^34,35^. When viewing a human face, observers gaze toward the right side of the face, which falls within the over-attended LVF^36–39^. This preference to examine the side of the face that falls within the LVF may further reflect the lateralised functions of the right hemisphere, which is not only dominant for visuospatial processing^29^, but also face processing^34,35^. Human infants, adults and rhesus monkeys, have all been shown to fixate on the left side of the human face, suggesting that the left gaze bias for human faces might be innate^37^. Furthermore, the visual scan paths displayed by the majority of individuals when examining a face also demonstrate an automatic LVF bias, which is not observed when the same individuals gaze at landscapes or symmetrical objects^40^. Finally, when given the opportunity to examine faces for an extended period of time, observers continue to spend significantly more time fixating the side of the face that falls within the LVF^37,38^. Therefore, when gazing at a group of faces, observers likely make more fixations toward the faces in the LVF, even over an extended time period. This left gaze bias for human faces is also reflected in the perceptual asymmetries shown by observers when making trait evaluations from faces^41,42^.

Observers not only spend longer exploring the right side of the face (i.e., the LVF)^38^, but base their trait evaluations of individuals upon the visual information present on the right side of the face^39,41,43,44^. Observers display a strong perceptual bias, which influences the perceived attractiveness of faces, such that the right side of the face is perceived to be more attractive than the left^41,43,45,46^, and faces presented entirely within the LVF are perceived as more attractive than faces presented in the RVF^46^. Burt and Perrett^41^ created chimeric faces, where one half of the face was highly attractive, while the other was unattractive. Participants were then presented with two identical faces, one which showed the attractive hemiface in the LVF, while the other was mirror reversed to show the attractive hemiface in the RVF. When asked which face was more attractive, observers displayed a strong bias to select the face with the attractive side presented in the LVF, despite the two faces being identical^41^. Furthermore, Dunstan and Lindell^43^ found that female faces were perceived to be more attractive when they showed the right cheek more prominently. However, when the same faces were mirror reversed, observers indicated that the left cheek was more attractive^43^. Crucially, the right side of the face is naturally viewed in the LVF, as is the left cheek when it is mirror reversed. Together these findings indicate a perceptual bias, such that faces are perceived to be more attractive when seen in the LVF.

Our aim was to investigate whether the LVF bias for face perception influences the magnitude of the cheerleader effect. We manipulated the spatial arrangement of the faces in the group image, such that the target face (i.e., the face being evaluated) would always be presented in the centre of the display, while the two distractor faces could be presented to the LVF, RVF, or on either side of the target. As attention is biased toward the LVF^27,28,39^, we expected that distractor faces within the LVF would be more salient to the observer than those in the RVF^37,39^. Increased visual exploration of the LVF could facilitate ensemble coding, as observers show greater accuracy in recalling complex visual scenes viewed in the LVF compared to the RVF^33^. Furthermore, the left gaze bias might also increase the perceived attractiveness of the distractor faces when they are seen in the LVF compared to the RVF^41,46^. Consequently, if the distractors in the LVF are perceived to be more attractive, the attractiveness of the ensemble average created from the group should also be increased. Under these conditions, the size of the cheerleader effect would increase, because the discrepancy between the attractiveness of the ensemble average and the individual face being evaluated should be greater. We predicted that the cheerleader effect would be larger when the distractor faces appeared in the LVF compared to the RVF.

## Experiment 1

### Method

#### Participants

Sixty-four participants from Flinders University (51 females, *M*_*age*_ = 25.58, *SD* = 8.42) received course credit for their participation. The Flinders Handedness Survey (FLANDERS) was used to assess participant handedness^47^. Scores on the FLANDERS can range from −10 (strongly left handed) to +10 (strongly right handed). Data from left- and mixed-handed participants (scores ≤ +5; *n* = 9) were excluded from analysis. Participants with a cheerleader effect score that was further than 3 *SD* from the condition mean (*n* = 3), who were visually impaired (*n* = 1), or who did not complete the task as instructed (*n* = 1), were also excluded from analyses. The final sample consisted of 50 strongly right-handed (*M* = 9.72, *SD* = 0.81) participants (41 females, *M*_*age*_ = 26.44, *SD* = 9.26). The procedures in the present research were approved by, and carried out in accordance with the guidelines of, the Social and Behavioural Research Ethics Committee of Flinders University.

#### Stimuli

**Figure 1:**
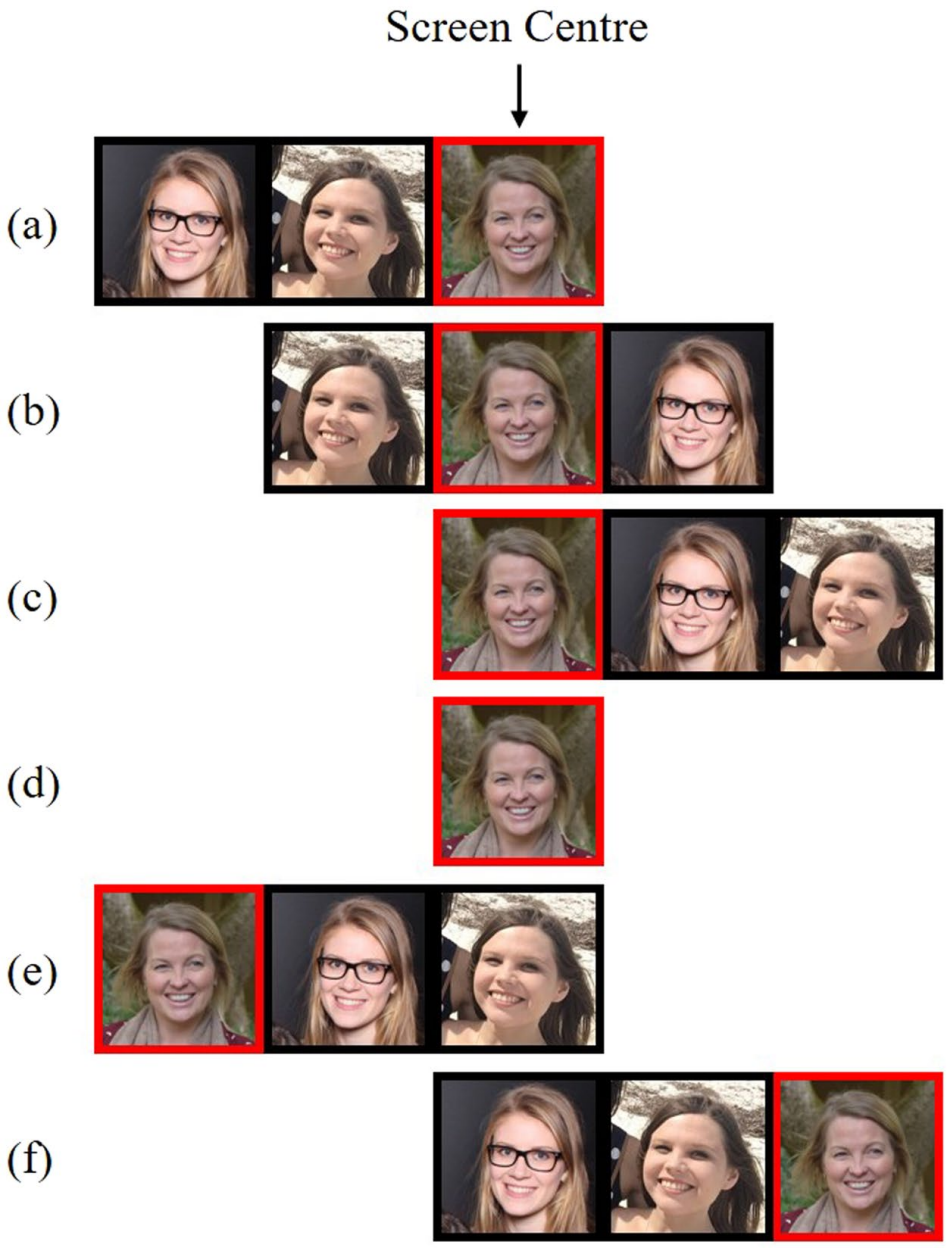
Example stimulus configurations in Experiment 1; (**a**) LVF distractors (**b**) BL distractors (**c**) RVF distractors (**d**) alone target (**e**) left dummy trial (**f**) right dummy trial. The target face (red frame) was presented in the centre of the display for critical trials (**a**,**b**,**c**,**d**).

Images of female faces were collected online, by querying an image search engine using the search term ‘Bridesmaids’^13,14^. To control for the possibility that individuals might pose differently in a group, all face stimuli originally came from photographs of groups. The faces of individual group members were closely cropped from the image to create individual portraits. Facial stimuli were selected that directly faced the camera, and had both eyes directed toward the camera. The majority of facial stimuli were estimated to be between 20–40 years old, showing joyous or happy expressions, and appeared to be of Caucasian ethnicity. Three individual portraits were shown horizontally side by side to create each group stimulus (see Fig. 1). Each individual portrait was 68 × 80 mm (7.78**°**, 9.15**°**) in size, and group images were 204 × 80 mm (23.06**°**, 9.15**°**). Stimuli were presented using E-Prime 2.0 (Psychology Software Tools, Pittsburgh, PA), interfaced with a 22” monitor (1680 × 1050) running at 60 Hz, which was positioned approximately 500 mm from the participant.

Target images were presented twice: once in a group with two distractors, and once alone as a portrait. Each distractor face appeared in only one group image. Target faces always appeared in the veridical centre of the display. On group trials, three distractor configurations were used: both distractors to the left of the target (LVF distractors), one on either side (bilateral distractors; BL), or both distractors on the right (RVF distractors). The distractor configuration for each target face was counterbalanced between-participants, such that each target face was rated with LVF, BL, and RVF distractors across participants. Importantly, each target face was shown with the same two distractor faces across participants, ensuring the attractiveness of the group remained constant^15^. The only difference between distractor conditions was the configuration of the distractors themselves.

Eighty-four faces were randomly selected from the stimulus set to appear as targets. All target faces were presented once as an individual portrait (*n* = 84) and once within a group (*n* = 84). The group image trials consisted of 28 trials for each distractor configuration (LVF, BL, RVF). Dummy group trials (*n* = 22), in which the ‘target’ was the far left or the far right face in the group and the distractors filled the two adjacent spaces (see Fig. 1e, 1f), were also included in the design to prevent participants from fixating on the central face, which was the target location on all critical trials. The dummy ‘target’ images (*n* = 22) were also shown in the alone condition to conceal their purpose. All dummy trials were discarded from analyses. In total, the experiment consisted of 212 trials, which were intermixed and randomised.

#### Procedure

Small groups of participants (*n* = 6–8) completed the experimental task individually. Informed consent was obtained from all participants prior to participation. Participants were asked to rate the attractiveness of the target face, which would be identified from the group image by a red frame appearing around the image. Six practice trials were completed to familiarise participants with all possible trial conditions (including dummy trials).

In group image trials, the image was initially presented for 2000 ms, during which time each face in the group was surrounded by a black frame. The target face was not identified from the group image during this initial free viewing phase, and participants were encouraged to examine each face in the group. The target face was then identified from the group image by a red frame, which surrounded the target face. The target was cued for 1000 ms, before all faces then disappeared from the display, and participants gave an attractiveness rating. During alone presentation trials, the target face was initially presented for 1000 ms with a black frame, which was replaced by a red frame for an additional 1000 ms. This presentation timing replicates that used by Walker and Vul (Experiment 4)^13^. The FLANDERS questionnaire was completed at the end of the experiment, to avoid priming participants about the lateralised nature of the task. The experiment took approximately 30 minutes to complete.

#### Analysis

Attractiveness judgments were made, via mouse click, along a visual analogue scale (width = 192 mm, 21.74°) that ranged from “Very Unattractive” (0%) to “Very Attractive” (100%). The spatial location of scale anchors was counterbalanced between-participants, such that half saw ‘Very Unattractive’ on the left and ‘Very Attractive’ to the right of the scale, while the other half of participants saw the anchors in the opposite orientation. Scale anchors were counterbalanced between participants to avoid stimulus-response compatibility effects, whereby participants might produce extreme responses on the side of the scale where the distractors appeared (i.e., lower attractiveness ratings for LVF distractors when “Very Unattractive” also appeared in the LVF)^48^. The dependent variable was the x-coordinate of the mouse click along the visual analogue scale, which was converted into a percentage of attractiveness prior to analysis.

The cheerleader effect refers to the change in attractiveness of the same face when seen in a group compared to alone^13^. As such, a cheerleader effect measure was calculated by subtracting the rating of attractiveness of targets when seen alone, from the attractiveness ratings made when the target faces were presented in each of the three group conditions. The three resulting change scores (one for each group condition), indicated as a percentage, the change in attractiveness experienced when a target face was seen in a group, with positive values indicating an increase in attractiveness.

#### Data Availability

The datasets generated and analysed in the current study are available in the Open Science Framework repository, [https://osf.io/rbg8q/].

### Results

**Figure 2:**
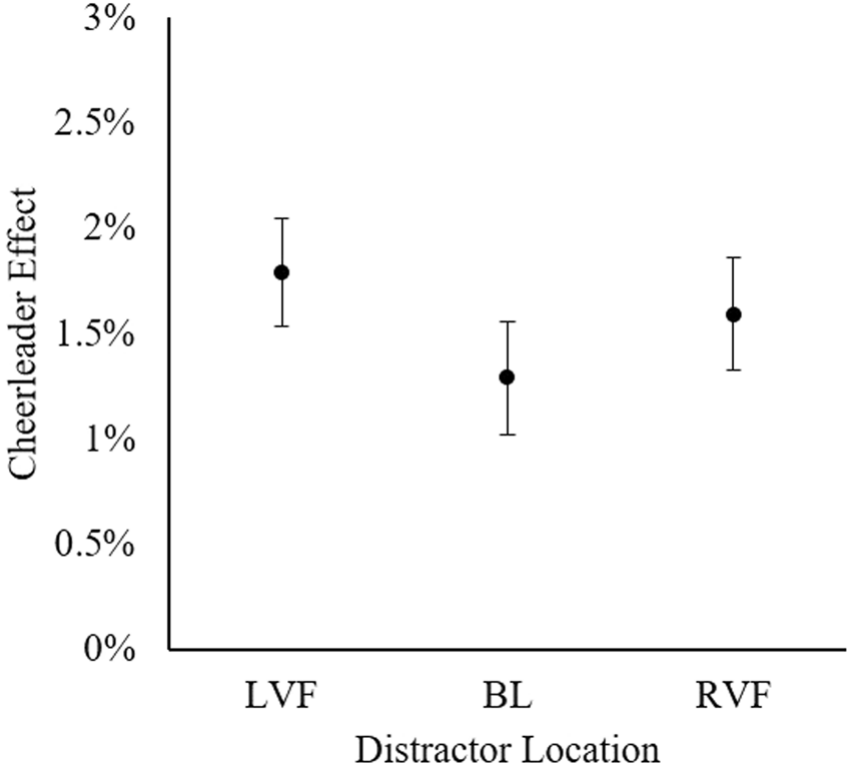
The cheerleader effect in each distractor configuration. Error bars represent within-subjects standard error^61^.

To first establish whether the cheerleader effect occurred in each group condition, we used three one sample *t*-tests to examine whether the change in attractiveness was statistically significant. Faces were perceived to be significantly more attractive in all group conditions: LVF, *t*(49) = 4.52, *95%* *CI* [1.00, 2.59], *p* < 0.001, *d* = 0.64; BL, *t*(49) = 3.24, *95%* *CI* [0.49, 2.09], *p* = 0.002, *d* = 0.46; RVF, *t*(49) = 4.45, *95%* *CI* [0.87, 2.31], *p* < 0.001, *d* = 0.63. The cheerleader effect was observed in each group condition, regardless of the spatial arrangement of the distractor faces (see Fig. 2).

A one-way within-participants analysis of variance (ANOVA) was used to investigate whether the strength of the cheerleader effect differed depending on the configuration of the distractor faces (LVF, BL, RVF). The effect of distractor configuration was non-significant, *F*(2, 98) = 0.610, *p* = 0.545, η^2^ = 0.012. Finally, we used a Bayesian ANOVA^49^ to investigate whether the observed data provided evidence in support of the null hypothesis. Our data were 8.96 times more likely to have occurred in the absence of an effect of distractor configuration, and therefore provide moderate evidence for the null hypothesis (BF_10_ = 0.11)^50^. Together, our results strongly suggest that the spatial arrangement of the distractor faces does not modulate the strength of the cheerleader effect.

### Discussion

Faces were perceived to be significantly more attractive when they appeared in a group, compared to when those same faces were seen alone. Our results offer the first replication of the cheerleader effect reported by Walker and Vul^13^. Furthermore, we observed the cheerleader effect in each group condition, suggesting that the effect is robust. Despite our prediction that LVF distractors would be more salient, the location of the distractor faces in the group images did not modulate the size of the cheerleader effect. A Bayesian analysis also indicated that the observed data were consistent with the null hypothesis. Indeed, the size of the cheerleader effect was similar regardless of the spatial position of the distractor faces around the target face.

A strength of our experimental design was that the target was always presented centrally. Any change to the size of the cheerleader effect could only be attributed to the spatial configuration of the distractor faces around the target face. However, this design could also be considered a limitation, as the target face was always presented centrally, rather than appearing within the LVF or RVF of the observer. The target face is the most important face in the group image, because it is the only face that the observer is required to evaluate. Perhaps it is the spatial location of the salient target face that modulates the size of the cheerleader effect, rather than the position of the irrelevant distractor faces. We conducted an exploratory follow up experiment, wherein the location of the target face was manipulated, in order to identify whether the cheerleader effect is sensitive to any manipulation of the spatial arrangement of the group.

## Experiment 2

Experiment 1 showed that the spatial arrangement of the *distractor faces* in the group did not modulate the size of the cheerleader effect. To investigate whether any change to the spatial composition of the group image might influence the cheerleader effect, we manipulated the spatial location of the *target face* in the group. Target faces were presented *furthest* from the centre of the display, either at the *far left* or *far right* of the group. Visual field differences might be revealed when the target face is shifted, because unlike the distractor faces, the observer is required to make an attractiveness evaluation of the target face. As the present study was exploratory in nature, we entertained the plausibility of multiple hypotheses (H_0_, H_1_, H_2_).

As illustrated by previous research, observers are likely to spend more time visually exploring the target face when it appears in the LVF^36,38,39^. Furthermore, the target might be perceived as more attractive when presented entirely within the LVF, compared to the RVF^41,43,46^. Consequently, when the target face is presented in the LVF, the group should be summarised to create an ensemble average that is more attractive than when the same target is presented in the RVF. As such, the increased attractiveness of the ensemble average in the LVF condition could result in a larger cheerleader effect, compared the RVF condition (H_1_).

Although it is possible that placing the target face within the LVF will increase the cheerleader effect (H_1_), it is also possible that positioning the target face within the LVF will produce a smaller cheerleader effect (H_2_), because observers are more likely to rely on the ensemble average under conditions of uncertainty^21^. When located in the LVF, observers are likely to spend more time examining the target face, compared to when it appears in the RVF^38^. If the observer has spent more time examining the target in the LVF, they may be less likely to rely on the ensemble average when recalling the attractiveness of the target face. In contrast, if fewer fixations are made to the target in the RVF, the observer may be uncertain about the attractiveness of the target face, and instead rely more on the attractiveness of the ensemble average. Therefore, it is also possible that the cheerleader effect will be smaller when the target face appears in the LVF and larger when the target appears in the RVF (H_2_).

Finally, it is also possible that the size of the cheerleader effect will not be influenced by the spatial position of the target face (H_0_). This pattern of results would be consistent with the evidence in favour of the null hypothesis reported in Experiment 1. Further evidence in favour of the null hypothesis would suggest that the spatial arrangement of the group does not influence the size of the cheerleader effect.

### Method

#### Participants

Sixty-six participants from Flinders University (44 females, *M*_*age*_ = 24.52, *SD* = 6.27) received course credit for their participation. As in Experiment 1, data from left- and mixed-handed participants were excluded (*n* = 6). All participants completed the task as instructed, and no participant data fell outside the 3 *SD* exclusion criterion used in Experiment 1. The final sample consisted of 60 strongly right-handed (*M* = 9.77, *SD* = 0.75) participants (40 females, *M*_*age*_ = 24.67, *SD* = 6.51).

#### Stimuli, Apparatus and Procedure

**Figure 3:**
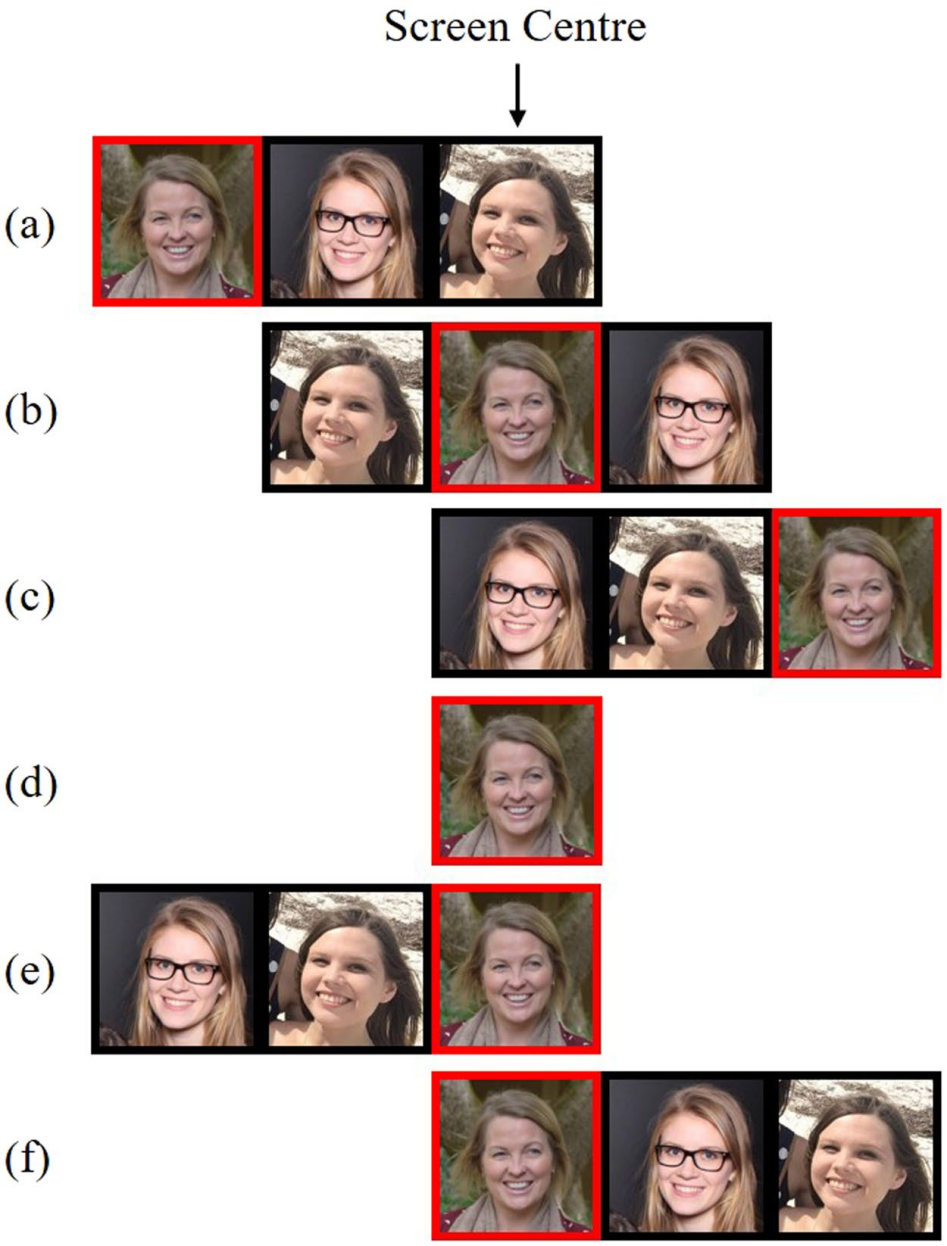
Example stimulus configurations for Experiment 2: (**a**) LVF target (**b**) Centre target (**c**) RVF target (**d**) alone target (**e**) left dummy trial (**f**) right dummy trial.

The apparatus, design, and procedure were identical to Experiment 1. The target faces and their accompanying distractor faces were also those presented in Experiment 1. In Experiment 2, we manipulated the spatial location of the *target face* within the group. Target faces could be presented in the LVF, Centre, or RVF (see Fig. 3). The location of each target image was counterbalanced between-participants, such that each target was rated in each of the three possible locations. Each target face was presented with the same two distractor faces in each group condition; only the position of the target face in the group differed between the group conditions. Dummy trials, which were not analysed, were included to prevent participants from fixating on the far ends of group images, where the target faces appeared during most critical trials. Dummy targets were presented in the centre of the display, with both distractors either to the left or right. In total, the experiment consisted of 212 trials, which were intermixed and randomised.

## Results

**Figure 4:**
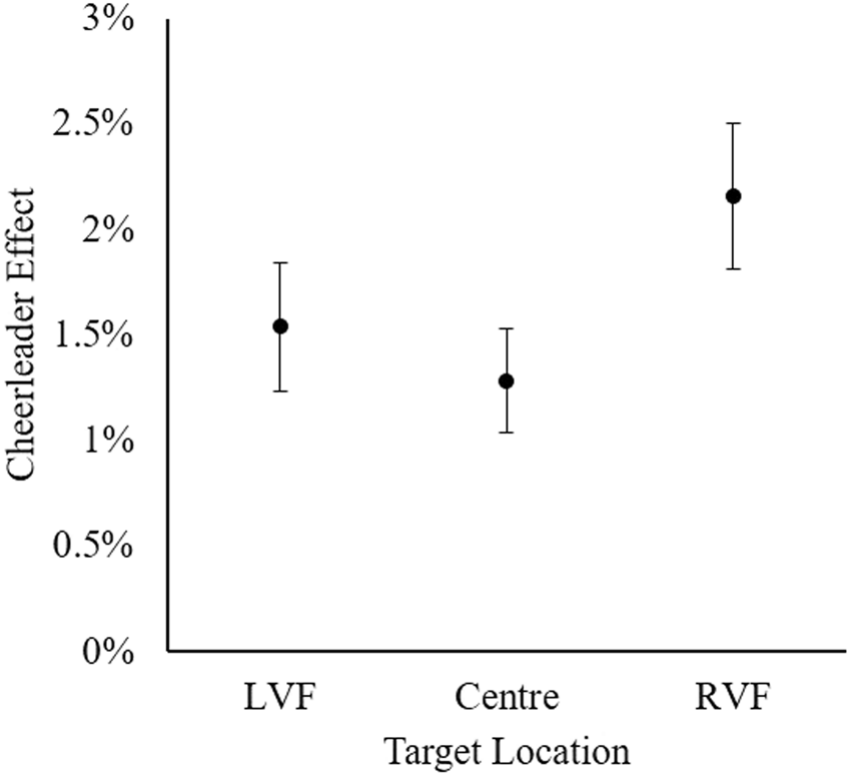
The cheerleader effect for each target location. Error bars represent within-subjects standard error^61^.

Three one sample *t*-tests were first used to examine whether the cheerleader effect was observed in each group condition. Faces were significantly more attractive in all group conditions: LVF, *t*(59) = 3.82, *95%CI* [0.73, 2.34], *p* < 0.001, *d* = 0.49; Centre, *t*(59) = 3.79, *95%CI* [0.61, 1.96], *p* < 0.001, *d* = 0.49; RVF, *t*(59) = 5.16, *95%CI* [1.32, 3.00], *p* < 0.001, *d* = 0.67. Once again, the cheerleader effect was observed in each group condition, regardless of the location of the target face in the group (see Fig. 4).

A one-way within-participants ANOVA was used to investigate whether the strength of the cheerleader effect was influenced by target location (LVF, Centre, RVF). As the assumption of sphericity was violated, Greenhouse-Geisser adjusted degrees of freedom are reported. The effect of target location was non-significant, *F*(1.75, 103.29) = 1.49, *p* = 0.230, η^2^ = 0.025. Finally, we used a Bayesian ANOVA^49^ to investigate whether the data provided evidence in support of the null hypothesis. Our data were 4.41 times more likely to have occurred in the absence of an effect of target location, and therefore provide moderate evidence for the null hypothesis (BF_10_ = 0.23)^50^. Taken together, our results strongly suggest that the location of the target face in the group image did not modulate the strength of the cheerleader effect.

### Discussion

The results of Experiment 2 showed a consistent cheerleader effect, regardless of the location of the target within the group. Our findings support the null hypothesis (H_0_), as the position of the target face in the group did not influence the strength of the cheerleader effect. Taken together, the results of Experiment 1 and Experiment 2 strongly suggest that the spatial configuration of the group does not influence the strength of the cheerleader effect.

## General Discussion

Across two experiments, we found strong evidence in support of the cheerleader effect^13^. In contrast to our predictions, the cheerleader effect was not influenced by perceptual or visual field biases, and occurred regardless of the spatial configuration of the group. The size of the cheerleader effect appears to be relatively consistent, such that attractiveness is increased within the range of 1.5–2%. Although the cheerleader effect is known colloquially^16^, scientific investigation has been limited^13^. Our findings show that the cheerleader effect is a robust phenomenon that can be observed using a relatively unconstrained set of images, collected from the internet. The cheerleader effect occurred in all group conditions, replicating Walker and Vul^13^, and extending upon their findings to show that the cheerleader effect is not modulated by the spatial configuration of the group image.

While the present study was not designed to directly test the proposed hierarchical encoding mechanism of the cheerleader effect, our findings are nonetheless consistent with the framework provided by Walker and Vul^13^. It is possible that the spatial configuration of the group did not influence the cheerleader effect, because ensemble coding serves to reduce perceptual redundancy, by rapidly encoding and summarising complex group displays^17^. Haberman and Whitney^20^ demonstrated that the average emotion shown in a group of 16 emotional faces could be accurately identified after being presented for only 500 ms. Therefore, ensemble coding can create accurate summary representations of much larger groups, which have been presented for less time, than the groups used in the present experiments. Furthermore, participants are sensitive to small changes in facial emotional expressions in large groups, without being able to identify the source of the change^51^, suggesting that encoding occurs without awareness for individual item locations within the set. The findings of Haberman and Whitney^51^ are consistent with previous research, which has shown that distractors are encoded and tracked, even though they are task-irrelevant and fall outside of the attended area of the visual display^52^. Thus, the results of the present study are consistent with an ensemble coding mechanism that can rapidly summarise large set sizes, and as such, is not influenced by visual asymmetries within a small set of images.

The hierarchal encoding framework offered by Walker and Vul^13^ is currently the only framework proposed to explain the cheerleader effect. Yet, the cheerleader effect shares many similarities with the *Group Attractiveness Effect*, whereby the attractiveness of a whole group of individuals is overestimated relative to the average attractiveness ratings of each individual group member^14^. The Group Attractiveness Effect is driven by selective attention towards the most attractive faces within the group, which results in an increased estimate of the attractiveness of the whole group^14^. The selective attention framework suggested by van Osch, *et al*.^14^ could also underlie the cheerleader effect, whereby the attractiveness of the target face is overestimated in a group, because attention is primarily directed towards the most attractive faces in the group^53^. As our data are potentially consistent with both accounts of hierarchical encoding^13^ and selective attention^14^, it is clear that future research is necessary to directly contrast the unique predictions of each model to determine the mechanism underlying the cheerleader effect.

Although imaging^35,54–56^ and behavioural^41,57–59^ studies indicate that the right hemisphere shows greater activation during face processing, bilateral and left hemisphere activation^35,60^ have also been reported. Proverbio, *et al*.^56^ found that females showed bilateral activation during face processing, whereas males showed asymmetric activation of the right hemisphere, suggesting that contradictory previous findings may be the result of sex differences in cerebral lateralisation. Similarly, Bourne^57^ found that males exhibited a stronger behavioural bias on a chimeric face task than females, further suggesting that males have stronger lateralisation of face processing than females, which manifests in stronger visual field asymmetries. As such, it is possible that the spatial configuration of the group images in the present study did not modulate the cheerleader effect because the majority of our participants were female. Although there were too few males in our sample to perform a reliable sex analysis, future research should consider whether the spatial configuration of the group image does modulate the cheerleader effect among males.

Our results clearly show that attractiveness judgments made for an individual face within a group are not the same as those made for the same face presented alone^13–15^. The presence of other faces in a group interferes with the perceived attractiveness of an individual, even when the individual target is clearly identified from the group. As the majority of previous research has examined trait perception of individual faces, future research of group social perception is vital^11^. For example, is the cheerleader effect a phenomenon that is specific to attractiveness judgments, or is the perception of other traits also influenced in a group scene? Other trait judgments, such as trustworthiness and competence, are strongly correlated with attractiveness^2^, and consequently may also be increased when an individual is seen within a group. However, the cheerleader effect might be unique to attractiveness judgments, because other traits are not as strongly related to facial averageness, and should not be systematically increased due to the average properties of the ensemble average of the group. Investigating whether the cheerleader effect extends to other traits is an exciting avenue for future research.

The cheerleader effect is a robust phenomenon, wherein faces appear more attractive in a group than alone. Our findings replicate those of Walker and Vul^13^, and show that the spatial configuration of the group does not influence the magnitude of the cheerleader effect. Ensemble coding of small groups might not be subject to the visual or perceptual biases that affect the perception of a single face. Our interpretation is consistent with previous research showing that group displays are encoded and summarised rapidly, and with little awareness of the individual group members. Our findings suggest that if you are looking to increase your own attractiveness, you could do so by appearing in a group, though you needn’t worry where you appear.
